# Synthesis of Monodisperse Silica Particles Grafted with Concentrated Ionic Liquid-Type Polymer Brushes by Surface-Initiated Atom Transfer Radical Polymerization for Use as a Solid State Polymer Electrolyte

**DOI:** 10.3390/polym8040146

**Published:** 2016-04-16

**Authors:** Takashi Morinaga, Saika Honma, Takeo Ishizuka, Toshio Kamijo, Takaya Sato, Yoshinobu Tsujii

**Affiliations:** 1 Department of Creative Engineering, National Institute of Technology, Tsuruoka College, 104 Sawada, Inooka, Tsuruoka, Yamagata 997-8511, Japan; morinaga@tsuruoka-nct.ac.jp (T.M.); saika@tsuruoka-nct.ac.jp (S.H.); takeo.ishizuka@live.jp (T.I.); kamijo@tsuruoka-nct.ac.jp (T.K.); 2 Global Research Center for Environment and Energy based on Nanomaterials Science (GREEN), National Institute for Materials Science (NIMS), 1-1 Namiki, Tsukuba, Ibaraki 305-0044, Japan; tsujii@scl.kyoto-u.ac.jp; 3 Institute for Chemical Research, Kyoto University, Uji, Kyoto 611-0011, Japan

**Keywords:** ionic liquid type polymer brush, ionic conductivity, surface initiated atom transfer radical polymerization, precise molecular weight analysis, solid state polymer electrolyte

## Abstract

A polymerizable ionic liquid, *N*,*N*-diethyl*-N*-(2-methacryloylethyl)-*N*-methylammonium bis(trifluoromethylsulfonyl)imide (DEMM-TFSI), was polymerized via copper-mediated atom transfer radical polymerization (ATRP). The polymerization proceeded in a living manner producing well-defined poly(DEMM-TFSI) of target molecular weight up to about 400 K (including a polycation and an counter anion). The accurate molecular weight as determined by a GPC analysis combined with a light scattering measurement, and the molecular weight values obtained exhibited good agreement with the theoretical values calculated from the initial molar ratio of DEMM-TFSI and the monomer conversion. Surface-initiated ATRP on the surface of monodisperse silica particles (SiPs) with various diameters was successfully performed, producing SiPs grafted with well-defined poly(DEMM-TFSI) with a graft density as high as 0.15 chains/nm^2^. Since the composite film made from the silica-particle-decorated polymer brush and ionic liquid shows a relatively high ionic conductivity, we have evaluated the relationship between the grafted brush chain length and the ionic conductivity.

## 1. Introduction

The high capacity lithium ion battery market has continued to rapidly expand as a large capacity source for automobiles and home energy storage, in addition to its traditional is as a small capacity battery for portable devices. However, accidental failure by fire or leakage issues of the electrolyte being used in a lithium ion cell have not yet been resolved. In order to solve this problem, growing attention has been paid to some solid electrolyte films made from ionic liquids [1,2] and poly(ionic liquids) materials [3,4]. Generally, an ionic liquid (IL) is defined as a salt having a melting point lower than 100 °C. They have a very low volatility, are flame retardant, and possess relatively high ionic conductivities [5]. Poly(ionic liquids), abbreviated as poly(ILs) in the following, can be formed into a thin film and it have attracted much attention as a nonflammable solid polymer electrolyte. They are obtained by polymerizing an ionic liquid-type monomer having a polymerizable substituent on the cation or the anion molecule of the ionic liquid. The first thorough studies of polymer electrolytes using poly(ILs) made by radical polymerization started around 2005 by Ohno *et al*. [6,7,8]. Valuable review [9,10] and feature articles have appeared [11] that detail the intensive studies on poly(ILs) during the last five years which have significantly expanded their research scope. Poly(ILs) have been prepared by a variety of approaches, including radical [3,12], and controlled radical polymerizations, such as atom transfer radical polymerization (ATRP) [13,14,15,16], reversible addition-fragmentation chain-transfer polymerization (RAFT) [17] and organotellurium-mediated living radical polymerization (TERP) [18]. Generally, poly(ILs) have good film formability and a relatively high heat resistance, however, they have a very serious problem, namely poor conductivity compared to ionic liquids.

Given this background, we have developed a totally new concept for fabricating a non-volatile, non-flammable solid electrolyte that is free from liquid leakage that has highly-conductive continuous ion-channel network [19]. It uses a surface initiated living radical polymerization (SILRP) grafting method applied to a new polymerizable ionic liquid. This process allows us to construct a crystal-like hybrid polymer/silica particles (PSiPs) having “concentrated” polymer brushes (CPBs) of ionic-liquid type polymers attached to the surface of a monodisperse silica surface.

Some of the important reports related to fine particles having CPBs have been published following Ohno’s report in 2002. They synthesized gold nanoparticles having a PMMA concentrated brush formed by surface initiated ATRP using a newly developed initiator which was fixed on the surface of the gold by the reductive cleavage reaction of a disulfide group [20]. Thereafter, Matsuno *et al.* succeeded to prepare fine magnetite particles having a polystyrene concentrated brush by surface initiated nitroxide mediated radical polymerization using a newly developed initiator. Fixing of the initiating group was accomplished by the condensation reaction of hydroxyl groups on the magnetite surface and phosphate groups of initiator [21]. An example of the synthesis of a concentrated polystyrene brush onto the silica fine particles has been reported by Kasseh *et al.* [22]. Our colleague Ohno, developed an ATRP immobilized initiator having a bonding group of trimethoxysilane, and succeeded in the synthesis of monodisperse silica fine particles modified with concentrated PMMA [23] and polyoxetane [24]. We have synthesized monodisperse silica fine particles having a concentrated ionic liquid type polymer brushes with the same initiator [19].

The particles decorated with poly(IL) brush form by three-dimensional self-assembly and contain a small amount of ionic liquid that functions as a plasticizer. Such a regular array of CPB-modified particles constitute a continuous 3D network array of polymer brushes, in which the chain ends of graft polymers concentrate in the boundary region. This arrangement is expected to enhance ion conduction as a result of segmental motion [19,25].

**Figure 1:**
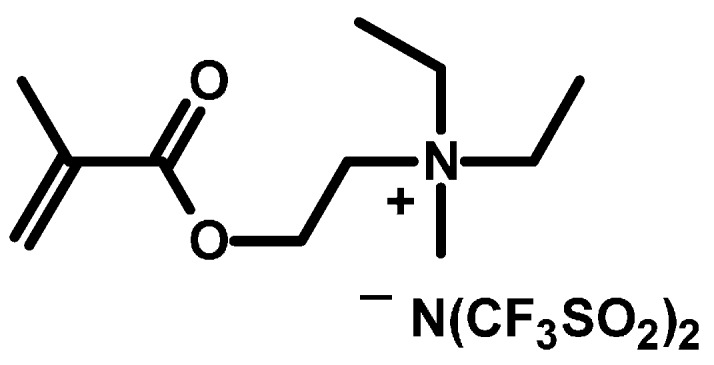
Chemical structure of *N*,*N*-diethyl*-N*-(2-methacryloylethyl)-*N*-methylammonium bis(trifluoromethylsulfonyl)imide (DEMM-TFSI).

The regular array structure and the high ionic conductivity of the PSiPs are derived from the specific nature of the concentrated poly(IL) polymer brush, so a precise characterization of the molecular weight and polydispersity index of the poly(IL) structure is required to better elucidate this new conduction mechanism and further improve the ionic conductivity of these systems. In this paper, we report data for the poly(IL) made by ATRP of the polymerizable ionic liquid, *N*,*N*-diethyl*-N*-(2-methacryloylethyl)-*N*-methylammonium bis-(trifluoromethylsulfonyl)imide (DEMM-TFSI, Figure 1). In the first part, we determine the exact molecular weight and its distribution by equilibrium dialysis and multi-angle laser light scattering coupled with GPC (GPC-MALLS). We also describe the surface-initiated ATRP of DEMM-TFSI, thereby producing monodisperse hybrid particles having a spherical silica core and a shell of well-defined ionic liquid polymer brushes. In particular, we have investigated the relationship of brush length and ionic conductivity. One interesting finding is effect on the regular sequence characteristics and ionic conductivity of PSiP’s of extending the brush chain length.

## 2. Materials and Methods

### 2.1. Materials

Ethyl 2-bromoisobutyrate (2-(EiB)Br, 98%) and 2,2′-Bipyridine (Bipy, 97%) were used as received from Nacalai Tesque Inc., Osaka, Japan. Copper(I) chloride (Cu(I)Cl, 99.9%) was purchased from Wako Pure Chemicals, Osaka, Japan. Copper(II) chloride (Cu(II)Cl_2_, 98%) was obtained from Nacalai Tesque Inc., Osaka, Japan. *N*,*N*-diethyl-*N-*(2-methoxyethyl)-*N*–methyl-ammonium bis(trifluoromethylsulfonyl)imide (DEME-TFSI) was purchased from Kanto Chemical Co., Inc. (Tokyo, Japan) and *N*,*N*-diethyl*-N*-(2-methacryloylethyl)-*N*-methylammonium bis(trifluoromethyl-sulfonyl)imide (DEMM-TFSI) was obtained from Toyo Gosei Co., Ltd., Tokyo, Japan, and purified by passing through a column of activated basic alumina to remove inhibitor. SiPs (SEAHOSTER, 20 wt % suspension of SiPs in ethylene glycol) were purchased from Nippon Shokubai Co., Ltd., Osaka, Japan. The average diameters of the SiPs were 130 (KE-E10), 290 (KE-E30), and 1550 nm (KE-E150) with relative standard deviations less than 10%, as measured by transmission electron microscopy. All other reagents were used as received from commercial sources.

### 2.2. Characterization

^1^H NMR (400 MHz) spectra were measured for a CD_3_CN solution of samples and are reported in ppm (δ) from the residual solvent peak (δ = 1.94) using a JEOL JEM-ECX400 spectrometer. Gel permeation chromatography (GPC) was performed on a Shodex GPC-101 high-speed liquid chromatography system equipped with a guard column (Shodex GPC KF-G, Showa Denko K. K., Tokyo, Japan), two 30 cm mixed columns (Shodex GPC KF-806L, exclusion limit = 2 × 10^7^), and a differential refractometer (Shodex RI-101) and multi-angle laser light scattering (MALLS) detectors (Wyatt Technology DAWN8^+^, Wyatt Technology, Santa Barbara, CA, USA). Acetonitrile/water (50/50 *v*/*v* mixture) containing 0.5 M acetic acid and 0.2 M NaNO_3_ were used as a eluents. The weight-average molecular weight was calculated from a calibration curve of PEG standards, and is abbreviated as *M*_w(PEG)_. The number-average molecular weight was calculated from the weight-average molecular weight determined by GPC-MALLS (*M*_w(MALLS)_) and MWD (*M*_w(MALLS)_/*M*_n(MALLS)_) obtained by the simple GPC analysis, and is abbreviated as *M*_n(MALLS)_.

Before RI increment (*dn*/*dc*) measurements, a solution of poly(DEMM-TFSI) in GPC solvent (polymer concentration; 2.0, 1.0, 0.5, 0.25 mg·mL^−1^ ) was dialyzed across a cellulose membrane tube (3500 Daltons molecular weight cutoff) in excess GPC solvent until the *dn*/*dc* value showed a constant value (equilibrium dialysis). The same batch of GPC solvent was used for the whole dialysis experiment because even possible experimental interbatch errors may affect RI increment measurements. The RI increment (*dn*/*dc*) was obtained by using a RI detector (Wyatt Optilab rEX, Wyatt Technology, Santa Barbara, CA, USA) for poly(DEMM-TFSI). The number-average molecular weight value was estimated as an absolute value, assuming a 100% initiation, from the monomer-to-initiator molar ratio and the conversion determined by ^1^H NMR, was abbreviated as *M*_n(theo)_. Thermogravimetric Analysis (TG) was performed with a Rigaku Thermo plus TG (Rigaku Corporation, Tokyo, Japan) at a heating rate of 10 °C·min^−1^. Infrared (IR) spectra were recorded on an IRAffinity-1 Fourier transform spectrometer (Shimadzu Corporation, Kyoto, Japan).

### 2.3. Living Radical Polymerization

The ATRP of DEMM-TFSI was carried out as follows. In a typical run, a Schlenk tube was charged with DEMM-TFSI (5.57 g, 11.6 mmol), 2-(EiB)Br (2.3 mg, 0.0116 mmol), Bipy (19.9 mg, 0.13 mmol), and acetonitrile (2.4 g), and the mixture was deoxygenated by purging with argon for 10 min. In a glove box purged with argon, Cu(I)Cl (5.2 mg, 0.052 mmol) or Cu(II)Cl_2_ (0.78 mg, 0.0058 mmol) was added to this mixture, and a three-way stopcock was attached to the Schlenk tube. In order to suppress the precipitation of copper chloride during the polymerization process, the reaction was carried out with a 10% excess of the ligand for the required molar amount of copper. Apart from the use of excess ligand, the stoichiometry of the reaction system is [DEMM-TFSI]_0_/[ethyl 2-bromoisobutyrate]_0_/[Cu(I)Cl]_0_/[Cu(II)Cl_2_]_0_/[2,2′-bipyridine]_0_ = 1000/1/4.5/0.5/10. The polymerization was carried out in an oil bath thermostated at 70 °C, and, after a prescribed time *t*, an aliquot of the solution was taken out for NMR measurement to estimate monomer conversion and for GPC measurement to determine the molecular weight and the distribution of the free chains produced in solution from the free initiator 2-(EiB)Br.

For preparation of silica particles (SiP) decorated with concentrated polymer brush, we used an initiator fixed SiP with an average diameter of 130 nm which was surface-modified in a mixture of ethanol/water/ammonia using a triethoxysilane having an ATRP initiating site, (2-bromo-2-methyl)-propionyloxyhexyltriethoxysilane (BPE), as reported previously [23]. In order to prepare the PSiP with Poly(IL) brush (*M*_n(theo)_ = *ca.* 25,000), the surface-initiated ATRP was carried out for 16 h at 70 °C in DEMM-TFSI (150.0 g, 312.2 mmol) containing 2-(EiB)Br (1.11 g, 5.67 mmol), Bipy (1.95 g, 12.5 mmol), Cu(I)Cl (0.450 g, 4.54 mmol), Cu(II)Cl_2_ (0.153 g, 1.14 mmol), and a suspension of initiator-coated SiPs (10.0 g) in acetonitrile (146.3 g) to yield SiP coated with poly(DEMM-TFSI) (PSiP). The polymerization reaction was also carried out with a 10% excess of the ligand. Apart from an excess of ligand, the stoichiometry of the reaction system is [DEMM-TFSI]_0_/[ethyl 2-bromoisobutyrate]_0_/[Cu(I)Cl]_0_/[Cu(II)Cl_2_]_0_/[2,2′-bipyridine]_0_ = 55/1/0.8/0.2/2. To prepare the PSiP with *M*_n(theo)_ = *ca.* 150,000 brush, the surface-initiated ATRP was carried out for 18 h at 60 °C in DEMM-TFSI (180.0 g, 374.6 mmol) containing 2-(EiB)Br (0.221 g, 1.135 mmol), Bipy (1.17 g, 7.49 mmol), Cu(I)Cl (0.303 g, 3.07 mmol), Cu(II)Cl_2_ (0.0458 g, 0.341 mmol), and a suspension of initiator-coated SiPs (10.0 g) in acetonitrile (118.4 g) to yield SiP coated with poly(DEMM-TFSI) (PSiP). The polymerization reaction was also carried out with a 10% excess of the ligand. Apart from an excess of ligand, the stoichiometry of the reaction system is [DEMM-TFSI]_0_/[ethyl 2-bromoisobutyrate]_0_/[Cu(I)Cl]_0_/[Cu(II)Cl_2_]_0_/[2,2′-bipyridine]_0_ = 330/1/2.7/0.3/6.

The molar ratio of the free-initiating group and fixed-initiating group which is located on the silica surface is calculated from the result of the graft density measurement was estimated [free-initiator]_0_/[fixed-initiator]_0_ = 98.9/1.1 and 94.0/6.0 for *ca.* 25,000 and 150,000 of *M*_n(theo)_, respectively. Since the fixed initiating site was a very small amount compared to the free (non-fixed) initiator, it would have no impact on the theoretical molecular weight calculation. The PSiP thus obtained was washed by five cycles of centrifugation and dispersion in acetonitrile to remove the unbound (free) polymer. The amount of grafted polymer was analyzed by thermogravimetry.

### 2.4. Preparation of Poly(DEMM-TFSI) by Radical Polymerization for GPC Analysis

To produce the calibration curve for the GPC-MALLS measurement of the molecular weights, the monomer DEMM-TFSI was polymerized by radical polymerization with AIBN and 3-mercapto-1-hexanol as an initiator and a chain transfer reagent (CTA), respectively in dimethylsulfoxide. In a typical run, a Schlenk tube was charged with DEMM-TFSI (1.0 g, 2.1 mmol), AIBN (0.01 g, 0.061 mmol), 3-mercapto-1-hexanol (1.0 mg, 0.0075 mmol), and DMSO 1.0 g and the mixture was deoxygenated by purging with argon for 10 min. The polymerization was carried out in an oil bath thermostated at 60 °C for 3 h. The concentration of the CTA was varied in three steps, 1.0, 25 and 65.4 mg. In all polymerization cases, a conversion of 100% was confirmed by ^1^H NMR spectroscopy.

### 2.5. PSiP/Ionic Liquid Composite Electrolyte Film Formation

Composite films of PSiP and DEME-TFSI, were prepared by casting their acetonitrle solutions with a composition (PSiP:IL = 75/25 *w*/*w* ratio) onto a cleaned substrate, followed drying and annealing at 90 °C for 36 h in vacuum, as reported previously [19].

## 3. Results and Discussion

### 3.1. ATRP of the Polymerizable Ionic Liquid, DEMM-TFSI

Compared to radical polymerizations carried out in other polar solvents, such reactions carried out in an ionic liquid lea to a significant increase in the normally observed *k_p_*/*k_t_* ratio [9,26]. For a variety of monomers, both the rates of polymerization and the molecular weights of the polymers produced were considerably higher in an ionic liquid. Furthermore, polymerizable ionic liquids, such as the DEMM-TFSI used here, result in higher molecular weight polymers in bulk radical polymerizations [3]. Difficulties associated with controlling the molecular weight might hinder development of these materials. Hence, we performed the polymerization of DEMM-TFSI under the molecular weight control made possible in the ATRP radical polymerization process. We have selected an acetonitrile that could completely dissolve both the monomer and polymer as a reaction solvent. Since the polymerization rate of ATRP trends to be faster in the polar solvents, a 2,2’-bipyridine having moderate activity was chosen as a ligand of a copper catalyst [27,28]. Furthermore, polymerization rate of this system has been optimized by adjusting the ratio of Cu(I)Cl/Cu(II)Cl_2_. In the ATRP experiment, the monomer conversion reached 90% after 5 h, which gave a polymer with the number-average molecular weight *M*_n(PEG)_ = 95,000 and a polydispersity index *M*_w(PEG)_/*M*_n(PEG)_ = 1.21 (calibrated by PEG standards).

**Figure 2:**
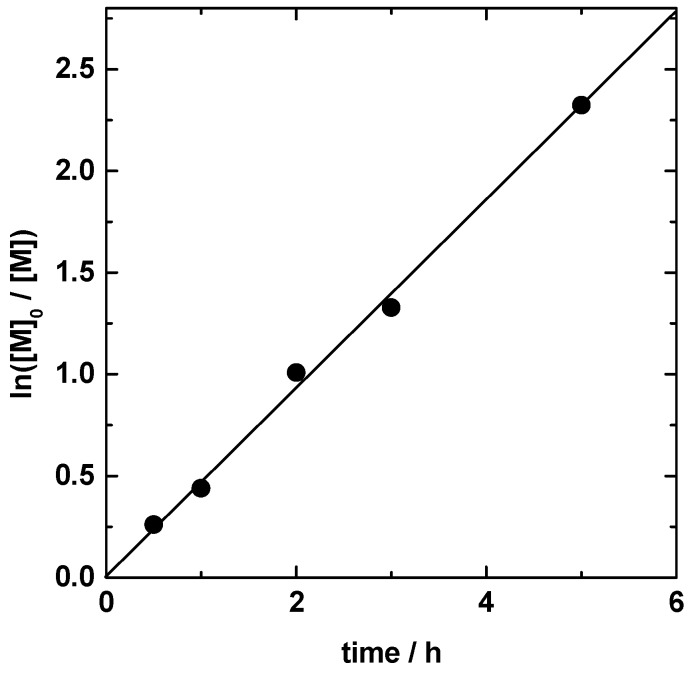
Plot of ln([*M*]_0_/[*M*]) *vs. t* for the solution polymerization of *N*,*N*-diethyl*-N*-(2-methacryloylethyl)-*N*-methylammonium bis(trifluoromethylsulfonyl)-imide (DEMM-TFSI, 70 wt %) in acetonitrile at 70 °C: [DEMM-TFSI]_0_/[ethyl 2-bromoisobutyrate]_0_/[Cu(I)Cl]_0_/[Cu(II)Cl_2_]_0_/[2,2′-bipyridine]_0_ = 1000/1/4.5/0.5/10.

Figure 2 shows the variation in ln([*M*]_0_/[*M*]) *vs.* polymerization time for the polymerization of DEMM-TFSI in acetonitrile at 70 °C with Cu(I)Cl/Cu(II)Cl_2_/Bipy as a catalyst. Almost full conversion was reached after 5 h and an almost linear first-order kinetic plot passing through the origin is seen until almost 100% conversion.

**Figure 3:**
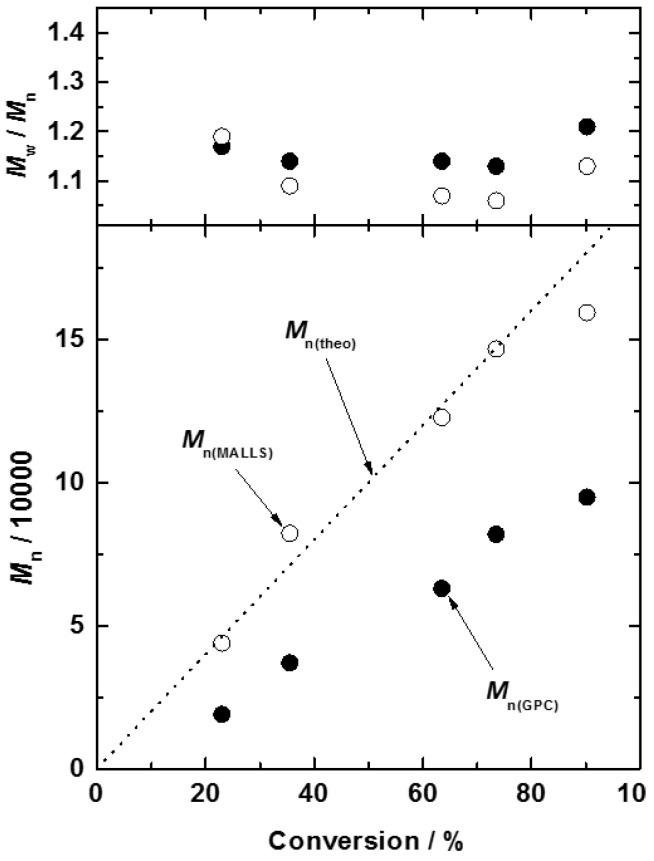
Evolution of number-average molecular weight (*M*_n_) and polydispersity index (*M*_w_/*M*_n_) of poly(DEMM-TFSI) estimated from GPC calibrated by standard poly(ethyleneoxide)s (filled circle) and by MALLS (open circle), as a function of monomer conversion for the solution polymerization of *N*,*N*-diethyl*-N*-(2-methacryloylethyl)-*N*-methylammonium bis(trifluoromethylsulfonyl)imide (DEMM-TFSI, 70 wt %) in acetonitrile at 70 °C: [DEMM-TFSI]_0_/[ethyl-2-bromoisobutyrate]_0_/[Cu(I)Cl]_0_/[Cu(II)Cl_2_]_0_/[2,2′-bipyridine]_0_ = 1000/1/4.5/0.5/10. The broken line represents the theoretical molecular weight (*M*_n(theo)_) values calculated with the initial molar ratio of DEMM-TFSI and the monomer conversion.

As shown in Figure 3, a linear increase in the number-average molecular weight determined by GPC spectroscopy with conversion is observed, indicating a constant number of propagating chains throughout the polymerization. The values of *M*_n_ estimated by PEG-calibrated GPC (*M*_n(PEG)_) deviated from the theoretical values *M*_n(theo)_ calculated from the initial molar ratio of DEMM-TFSI to the initiator. This may be due to the difference in the hydrodynamic volume of the PEG and the poly(DEMM-TFSI). In order to confirm this and obtain a precise molecular weight of ionic liquid type polymer, a GPC calibration curve for poly(DEMM-TFSI) was made using GPC-MALLS measurements. For producing the calibration curve for GPC-MALLS, it was known that a lower polymer concentration gives a more accurate molecular weight value in the static light scattering (SLS) measurement when working at the detection limits of the SLS. In order to obtain accurate molecular weights over a wide eluent volume, use of a polydisperse polymer sample is convenient. The molecular weights determined by SLS are calculated on the basis of the Zimm plot measured in the dilute polymer concentration region. In the measurement of GPC using a monodisperse polymer as a standard sample, it is often not possible to maintain the infinite dilution state of polymer concentration in the eluent, because the polymer concentration in the effluent rapidly increases at a specific elution time. Therefore, to carry out the SLS measurements with high accuracy in a large analysis area, it is desirable to use a polydisperse polymer sample as a standard sample. We have used polydisperse poly(DEMM-TFSI) which covered the molecular weight range of GPC columns prepared by a simple radical polymerization with charge transfer agent (CTA).

**Figure 4:**
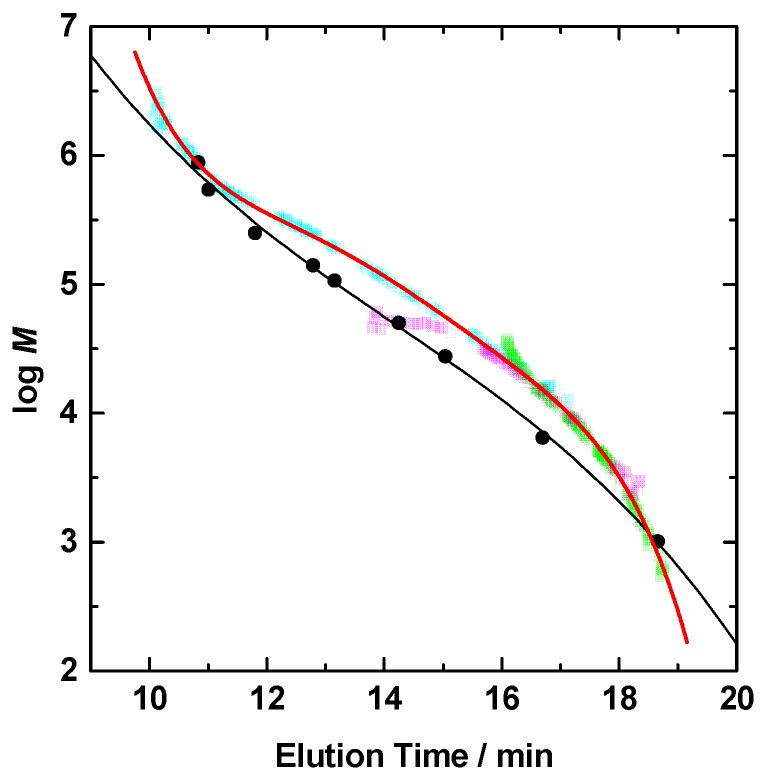
Calibration curves for the molecular weight calculation estimated with standard poly(ethyleneoxide)s (black circle) and molecular weights of poly(DEMM-TFSI) obtained from GPC-MALLS (red line). Molecular weights of poly(DEMM-TFSI) was calculated by the intercept of Zimm plots from the each SLS measurement at respective elution times (blue, pink, and green dots).

The molecular weight and the polydispersity of molecular weight of the polymer used for calibration curve for GPC-MALLS are summarized in Table 1. To know the exact molecular weight of poly(DEMM-TFSI), *dn*/*dc* was measured with a differential refractometer. Poly(IL) can be approximated to be the state of infinite dilution in the column during the measurement. In order to accurately measure the *dn*/*dc* in that state, we had to reproduce the salt concentration equilibrium of the infinite dilution condition by dialysis of a solution with an excess of the GPC eluent at each polymer concentration. A 0.2 wt % of poly(DEMM-TFSI) solution in GPC solvent (50% *v*/*v* mixture of acetonitrile/water containing 0.5 M acetic acid and 0.2 M NaNO_3_), was dialyzed to be in an equilibrium condition in an excess amount of natural GPC solvent. The *dn*/*dc* value of the dialyzed poly(DEMM-TFSI) solution was 0.197, higher than that of the solution before dialysis (0.170). The value obtained in this way can be said to be the correct *dn*/*dc* in the GPC eluent for poly(DEMM-TFSI). According to the *dn*/*dc* value, we could then make an accurate GPC calibration curve for poly(DEMM-TFSI) by GPC-MALLS measurements, as shown in Figure 4.

We have proposed that the use of three polydisperse poly(DEMM-TFSI)s with different molecular weights allows us to almost completely cover the analytical range of the GPC column. At the top and tail parts of each GPC trace, the accuracy of SLS would be low due to the low polymer concentration in the eluent. However, we can make a single calibration curve for poly(DEMM-TFSI) covering a wide molecular weight range by connecting the measurement portions made with high accuracy. The data shown in Figure 4 suggests that there is a major difference in the hydrodynamic volume between PEG and poly(DEMM-TFSI). Compared to the calibration curve made from PEG standards in the range of 12 to 17 min of elution time which corresponds to a analytical range for the GPC column and a required molecular weight range in the analysis for our poly(IL)s , the overall poly(DEMM-TFSI) molecular weights are high, and has a low-pitched gradient means indicating that the true molecular weight distribution is lower than that from the PEG calibration. For instance, when the by PEG calibration produced a number-average molecular weight *M*_n_ = 82,000 and a polydispersity index *M*_w_/*M*_n_ = 1.13 for poly(DEMM-TFSI) , the same sample analyzed using a GPC-MALLS calibration revealed values of *M*_n_ = 145,000 and *M*_w_/*M*_n_ = 1.06. As shown in Figure 3, the GPC-MALLS calibrated *M*_n_ value (*M*_n(MALLS)_) was larger than the *M*_n(PEG)_ values and closer to the *M*_n(theo)_ value. This allowed us to adopt the theoretical *M*_n_ values in the following discussion. The *M*_w_/*M*_n_ ratio was consistently small (*M*_w(MALLS)_/*M*_n(MALLS)_ = 1.19–1.06) from the first stage of the polymerization to the end. These data indicated that the molecular weight controlled synthesis of poly(DEMM-TFSI) with a narrow *M*_w_/*M*_n_ ratio can be successfully performed by ATRP of DEMM-TFSI. We, thus, succeeded in characterizing the ionic liquid-type polymer using GPC-MALLS, which produces values corresponding to the theoretical *M*_n_ value.

### 3.2. Surface-Initiated ATRP of Polymerizable Ionic Liquid, DEMM-TFSI

**Figure 5:**
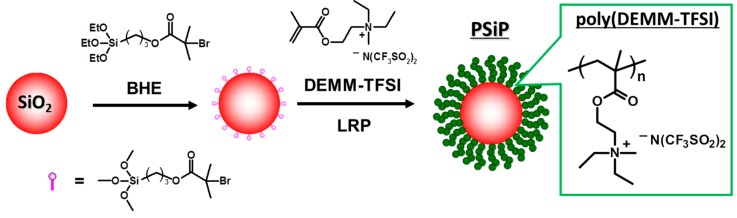
Schematic representation of the synthesis of SiP grafted with poly(DEMM-TFSI) brushes by surface-initiated ATRP.

The surface-initiated ATRP of DEMM-TFSI proceeded in the same living manner as was the case of polymerization without initiator-coated SiP, providing poly(DEMM-TFSI)-SiP. In a typical run, the solution polymerization of DEMM-TFSI (50 wt %) in acetonitrile with the initial molar ratio of [DEMM-TFSI]_0_/[ethyl 2-bromoisobutyrate]_0_/[Cu(I)Cl]_0_/[Cu(II)Cl_2_]_0_/[2,2′-bipyridine]_0_ = 55/1/0.8/0.2/2 in the presence of initiator-coated SiP of diameter 130 nm was carried out at 70 °C for 16 h, and gave a monomer conversion of 98% which translates into a poly(DEMM-TFSI) graft with *M*_n(theo)_ = 25,600. The schematic preparation procedure is shown in Figure 5.

Thermogravimetric analysis (TGA) for the poly(DEMM-TFSI)-SiPs prepared above was carried out to estimate the amount of polymer grafted on the SiP. With the estimated amount of polymer, 8.9 wt % against whole amount of poly(DEMM-TFSI)-SiPs, the graft density was calculated to be 0.15 chains/nm^2^ using the known density and surface area of the SiP and the *M*_n_ of the graft polymer. We also confirmed the quantity of polymer brush in the PSiP measured by the TGA was directly proportional to the theoretical *M*_n_ of polymer brush. At this time, *M*_n_ of the brush polymer has been assumed to be equal to the free polymer molecular weight obtained from the free initiator. We have confirmed that the molecular weight and polydispersity index of grafted PMMA and polyoxetane brush by the cut-out of brush polymer by silica dissolution using HF were almost equal to the values of free polymer [23,24]. A research group at Kyushu University in an experiment aimed at synthesizing a polymer brush of polystyrene and Poly(3-vinylpyridine) showed that the *M*_n_ and *M*_w_/*M*_n_ of the fixed brush polymer was almost same as the value of the free polymer by cleaving the polymer brush from the particle [21]. From such reports, the assumption has been widely accepted that the molecular weight and polydispersity of fixed polymer brushes on the surface and the free polymer are substantially equal. In this study, we had to estimate the graft density of PSiPs by using the molecular weight of the free polymer, since decomposition of poly(DEMM-TFSI) brush might occur from the application of HF. The surface occupancy σ* (dimensionless graft density), reduced by the cross-sectional area of the fully-stretched polymer chain, estimated using a bulk density of 1.4 g/cm^3^ for poly(DEMM-TFSI), was 0.34. This σ* value places the ionic-polymer brush prepared in this study in the CPB regime. We also attempted graft polymerization with another two SiPs with diameters of 290 and 1550 nm, respectively. The results are summarized in Table 2.

**Figure 6:**
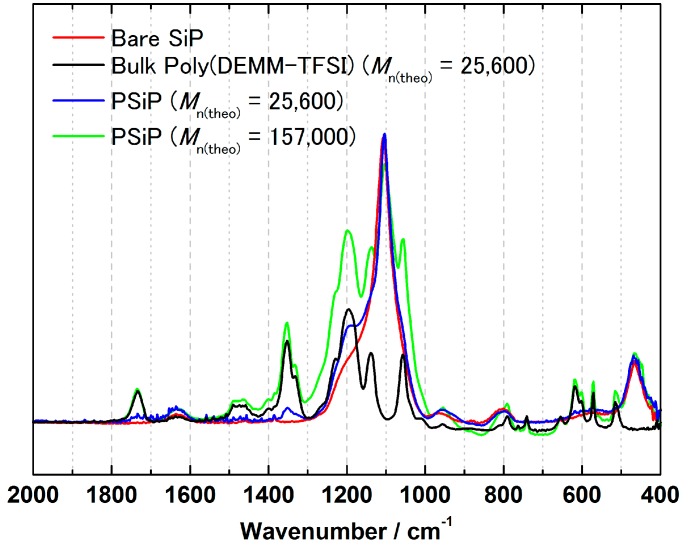
Fourier transform-infrared spectra of the products at each step for the fabrication of the PSiPs. The blue and green lines correspond to the hybrid particles after surface-initiated ATRP with number-average molecular weights of the poly(DEMM-TFSI) grafted onto SiPs are 25,600 and 157,000, respectively. The red and black lines corresponds to the pure products of SiP and poly(DEMM-TFSI), respectively. The diameter of the SiP core was 130 nm.

Figure 6 shows the IR spectra of the silica particles with and without brush polymer and bulk poly(DEMM-TFSI) as KBr pellets. The peak intensity at 1730 and at around 1375 cm^−1^, which are assigned to the C=O stretching of the ester bond of the methacrylates and to the CH_3_ deformation against the peaks around 800 and 1100 cm^−1^, which are derived from the SiP core, increased after the surface-initiated ATRP, indicating that the propagation reaction of the grafted polymer chain had taken place.

We also confirmed that we could prepare a concentrated ionic liquid-polymer brush at a graft density of 0.13 chains/nm^2^ using a flat silicon wafer substrate. At this time, for graft density calculation, we used the dry film thickness of polymer brush measured by the ellipsometry (8.4 nm at *M*_n(theo)_ = 61,000) and the molecular weight, which was assumed to be equal to the molecular weight of free polymer. The fact that the brush density was constant regardless of the silica particle size and the shape of the substrate under the same polymerization conditions suggests that the brush density depends on the density of the initiation site. We are planning more detailed experiments on this topic. In any case, the polymerization was well controlled in all cases, as indicated by the low polydispersity of the graft polymers and the high graft density. Our brush forming method is able to import a concentrated ionic liquid-polymer brush onto the surface of various substrates. Suspending the grafted PSiP material in DEME-TFSI at a certain concentration produced an iridescent dispersion suggesting the formation of a colloidal crystal. We able to make a film with sufficient physical strength to be free-standing by simply casting it from a PSiP/IL mixture (colloidal crystal) diluted with a volatile mixed organic solvent such as acetonitrile and propylene carbonate solution. A mixture that was 75 wt % PSiP and 25 wt % IL (31 vol %) gave a solid electrolyte film with a high ionic conductivity [19].

**Figure 7:**
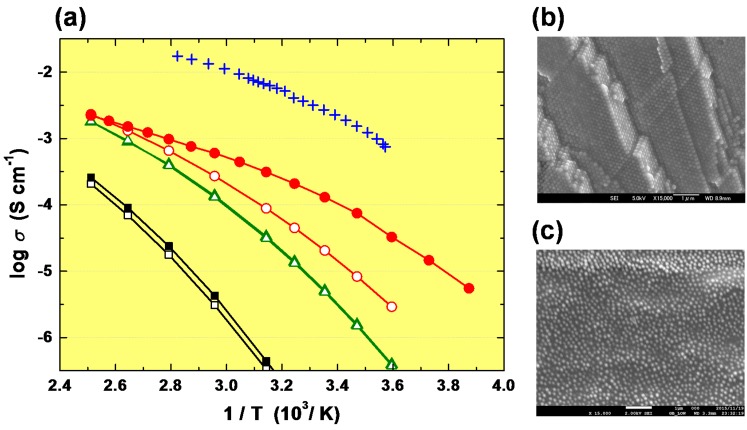
Arrhenius plots of ionic conductivity σ (**a**) for PSiP/IL solid, IL(DEME-TFSI) liquid and bulk poly(DEMM-TFSI) (*M*_n(theo)_ = 25,600, 157,000), together with SEM images (**b**,**c**) of fractured surfaces of PSiP/IL-solid electrolyte observed at a magnification of 15,000×. In the (**a**), **red** open circle, PSiP/IL solid with polymer brush of *M*_n(theo)_ = 157,000; **red** filled circle, PSiP/IL solid with polymer brush of *M*_n(theo)_ = 25,600; **blue** cross, DEME-TFSI (ionic liquid); **black** filled square, bulk poly(DEMM-TFSI) of *M*_n(theo)_ = 25,600; black open square, bulk poly(DEMM-TFSI) of *M*_n(theo)_ = 157,000; **green** filled triangle, bulk poly(DEMM-TFSI) of *M*_n(theo)_ = 25,600 including 25% of ionic liquid, DEME-TFSI; **green** open triangle, bulk poly(DEMM-TFSI) of *M*_n(theo)_ = 157,000 including 25% of ionic liquid, DEME-TFSI. SEM image of PSiP/IL solid with polymer brush of *M*_n(theo)_ = 25,600 for (**b**) [19] and PSiP/IL solid with polymer brush of *M*_n(theo)_ = 157,000 for (**c**).

The temperature dependence of the ionic conductivity for PSiP/IL composite films, ionic liquid (DEME-TFSI), and poly(DEMM-TFSI) bulk film with, and without, ionic liquid as a plasticizer, and scanning electron microscopy images of solid electrolytes are shown in Figure 7. The PSiP composite film with a longer ionic liquid concentrated brush (red open circle) did not show a high ionic conductivity, rather it showed a similar conductivity the bulk film of poly(DEMM-TFSI) that included 25 wt % of ionic liquid as a plasticizer (green open and filled triangle). They exhibit lower ionic conductivity regardless of the molecular weight of the polymer than the PSiP/IL solid, and they had a similar activation energy of ion conduction to the bulk film containing no plasticizer. The bulk polymer showed the lowest ionic conductivity among measured samples, regardless of the molecular weight. Since the surface of the spherical core silica particle has a curvature, a longer brush will allow a larger free space for movement of the tip of the stretched polymer brushes. The surface made by the polymer brush with a molecular weight of 157,000 no longer has a concentrated density [29]. Where entanglement and interpenetration of the brush occurs in the tip portion of the brush, the material will appear to be of the same nature as the bulk polymer.

On the other hand, PSiP having a relatively short polymer brush on the surface which has high brush density showed a regular three-dimensional array (Figure 7b). All particles are three dimensionally-aligned at intervals of approximately twice the distance of the brush length. It is likely that a regular array of CPB-modified particles will form a continuous three-dimensional brush network with the chain ends concentrated at the boundary. In this case, the segmental motion might be expected to lead to increased ionic conduction. The PSiP with a short brush (*M*_n(theo)_ = 25,600), red filled circle in Figure 7a has a higher ionic conductivity than PSiP with a longer brush. The SEM images of PSiP with the longer polymer brush did not reveal any apparent three-dimensional ordered array of PSiP, the PSiPs are arranged at random in the polymer matrix in Figure 7c. The particles are embedded in a polymer matrix, the particles being connected by bulk polymer. Hence, we can say that in order to exhibit a high ionic conductivity and a regular arrangement of particles, the brush concentration of PSiP must remain concentrated. This result suggests that we should use a PSiP having a relatively short brush in order to produce high ionic conductivity. Currently we are searching for a composition that combines a very high ionic conductivity and good film strength.

## 4. Conclusions

A polymerizable ionic liquid having a methacryl functional group as part of the ionic liquid cation, DEMM-TFSI, was successfully polymerized by ATRP with molecular weight control of a target poly(DEMM-TFSI) up to about 400 KDa (including a polycation and an counter anion). We established a method of characterizing the ionic liquid-type polymer using GPC-MALLS, which produces values corresponding to the theoretical *M*_n_ value. The DEMM-TFSI monomer was successfully polymerized by surface-initiated ATRP to produce SiPs grafted with well-defined poly(DEMM-TFSI) brushes in a concentrated regime. A PSiP with relatively long polymer brushes does not show a high ionic conductivity due to entanglement of the polymer making it impossible to form effective ion conducting paths in the matrix. To the contrary, a PSiP with a relatively short polymer brush showed a good ionic conductivity resulting from the formation of a three-dimensional array structure and a good conductive path in the solid electrolyte matrix.

## Figures and Tables

**Table 1:** Characteristics of poly(DEMM-TFSI) made by radical polymerization for GPC calibration curve.

Entry	[AIBN]/[CTA] (by mol fraction)	*M*_n(MALLS)_	*M*_w(MALLS)_/*M*_n(MALLS)_
1	1/0.12	754,000	2.25
2	1/3	47,000	1.62
3	1/8	13,000	1.95

**Table 2:** Characters of silica particles grafted with concentrated poly(DEMM-TFSI) brush.

**Entry**	Diameter of Silica Particle (nm)	Conv. (%)	*M*_n(theo)_	*M*_w(PEG)_/*M*_n(PEG)_	Graft Density (Chains/nm^2^)
1	130	99	25,600	1.11	0.15
2	130	99	157,000	1.09	0.21
3	290	98	66,000	1.17	0.15
4	1,550	90	285,000	1.17	0.17

## Abbreviations

The following abbreviations are used in this manuscript:

DEMM-TFSI	*N*,*N*-diethyl*-N*-(2-methacryloylethyl)-*N*-methylammonium bis(trifluoromethyl-sulfonyl)imide
ATRP	Atom transfer radical polymerization
GPC	Gel permeation chromatography
SiP	Silica particle
Poly(IL)	Poly(ionic liquid)
IL	Ionic liquid
RAFT	Reversible addition-fragmentation chain-transfer
TERP	Organotellurium-mediated living radical polymerization
PSiP	Polymer/silica particle
CPB	Concentrated plymer brush
MALLS	Multi-angle laser light scattering
DEME-TFSI	*N*,*N*-diethyl-*N-*(2-methoxyethyl)-*N*–methyl-ammonium bis(trifl uoromethylsulfonyl)-imide (DEME-TFSI)
DSC	Differential scanning calorimetry
CTA	Charge transfer agent
PEG	Polyethylene glycol
SLS	Static light scattering
AIBN	Azobisisobutyronitrile
